# Concepts for nondestructive and depth-resolved X-ray residual stress analysis in the near-surface region of nearly single crystalline materials with mosaic structure

**DOI:** 10.1107/S1600576720014016

**Published:** 2021-02-01

**Authors:** Andreas Hollmann, Matthias Meixner, Manuela Klaus, Christoph Genzel

**Affiliations:** a Helmholtz-Zentrum Berlin für Materialien und Energie, Germany

**Keywords:** residual stress, X-ray diffraction, depth-resolved analysis, mosaic crystals

## Abstract

Two evaluation concepts for nondestructive depth-resolved X-ray residual stress analysis in the near-surface region of materials with cubic symmetry and nearly single crystalline structure are introduced by simulated examples. Both concepts are based on the same data acquisition strategy, which consists in the determination of lattice-spacing depth profiles along the 〈*hkl*〉 poles by stepwise sample rotation around the scattering vector. Segmentation of these profiles parallel to the sample surface provides the lattice strain state as a function of depth. The first evaluation concept extends the crystallite group method developed for materials with pronounced crystallographic texture by the feature of depth resolution and can be applied to samples with arbitrary orientation. The second evaluation concept, which adapts the linear regression approach of the sin^2^ψ method for the case of single crystalline materials, is restricted to samples with (001) orientation. The influence of the strain-free lattice parameter *a*_0_ on residual stress analysis using both evaluation concepts is discussed on the basis of explicitly derived relations.

## Abstract 2

Two data evaluation concepts are proposed for nondestructive and depth-resolved X-ray residual stress analysis by means of energy-dispersive diffraction on materials featuring cubic symmetry and a nearly single crystalline structure.

## 1. Introduction   

X-ray residual stress analysis (XSA) has a long tradition whose roots reach back to the 1930s [see, for example, Noyan & Cohen (1987 ▸) and Hauk (1997 ▸)]. Since then numerous methods have been developed. The majority of them target the analysis of the residual stress state in polycrystalline materials with an almost random orientation distribution of the crystallites, which can be regarded as quasi-isotropic on a macroscopic length scale. The elastic anisotropy of the crystallites the material consists of is taken into account by the diffraction elastic constants (DEC), 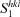 and 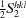. They are independent of the measurement direction (φ, ψ) in the sample reference system but depend on the analyzed crystal lattice planes *hkl*. The DEC can be determined experimentally or calculated on the basis of different grain interaction models such as those proposed by Voigt (1910 ▸) (homogeneous strain), Reuss (1929 ▸) (homogeneous stress), or Eshelby (1957 ▸) and Kröner (1958 ▸) (elastic polarizability).

Materials with preferred crystallographic orientation behave as elastically anisotropic on a macroscopic length scale as well. Diffraction stress analysis of such materials requires a treatment which differs from that to be applied to specimens featuring preferred orientation of the crystallites (Welzel & Mittemeijer, 2003 ▸). The relationship between the lattice strain 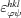 obtained in some direction (φ, ψ) and the stress components σ_*ij*_ can be described by means of stress factors *F*_*ij*_(φ, ψ, *hkl*) (Dölle & Hauk, 1978 ▸, 1979 ▸). Their calculation from the elastic single crystal constants requires knowledge of the orientation distribution function as weighting factor when averaging over the reflecting crystallites in the measurement direction (Behnken & Hauk, 1991 ▸; Behnken, 2003 ▸).

Very sharp crystallographic textures allow crystallites with almost identical orientation to be grouped together and treated as single crystals for stress analysis. The relationship between the lattice strains and the components of the residual stress tensor is then given by the generalized Hooke law, with the fourth-rank tensors of the elastic compliances and stiffnesses, ^4^ 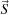 and ^4^ 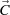, respectively, acting as connecting factors (Nye, 1985 ▸). On the basis of this approach the crystallite group method was introduced by Willemse *et al.* (1982 ▸) and applied to residual stress analysis in cold-drawn wires. Later, the concept was further developed (Hauk & Vaessen, 1985 ▸; Hauk & Oudelhoven, 1988 ▸) and used to determine the residual stress state in plates with a pronounced rolling texture as well as in materials with strong fiber texture (Baron & Hauk, 1988 ▸).

A limitation of most XSA methods developed so far for materials with pronounced texture is that they only provide average values of the residual stress state at the near-surface region irradiated by the X-ray beam. The number of measurement directions is restricted to the 〈*hkl*〉 poles of the texture, which prevents a continuous variation of the information depth τ by changing the inclination angle ψ between the surface normal and the measurement direction. Therefore, approaches for residual stress gradient analysis suitable for materials with almost random orientation distribution of the crystallites such as the universal plot method (Ruppersberg *et al.*, 1989 ▸, 1991 ▸), the low incidence beam angle diffraction method (Van Acker *et al.*, 1994 ▸; Mohrbacher *et al.*, 1996 ▸), the multi-wavelength method (Eigenmann *et al.*, 1990 ▸) and the mixed Ω–Ψ mode technique (Dümmer *et al.*, 1999 ▸; Erbacher *et al.*, 2008 ▸) cannot be applied. They are based on the measurement principle of the 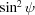 method (Macherauch & Müller, 1961 ▸), according to which evaluable diffraction lines are available in any measurement direction.

The aim of the present work is to extend the XSA methodology for materials with nearly single crystalline structure by the possibility of depth resolution. The data acquisition strategy proposed to achieve depth resolution is based on the scattering vector method (Genzel, 1994 ▸). It allows the determination of lattice-strain depth profiles 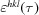 along the 〈*hkl*〉 poles of strongly textured materials by stepwise sample rotation around the scattering vector (Genzel, 1999 ▸; Genzel *et al.*, 1999 ▸). Segmentation of these profiles parallel to the surface provides sets of strain data which form the basis of two evaluation concepts proposed in this paper for the determination of the residual stress state as a function of depth. Both evaluation concepts use the stress factors to relate the lattice strains to the stress components to be determined. The first concept follows the crystallite group method by calculating the unknown stress components from sets of overdetermined systems of equations. Its applicability is not restricted to a special crystallographic orientation of the sample. The second concept adapts the linear regression approach of the 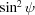 method. Since this concept requires a sufficient number of 〈*hkl*〉 poles along fixed azimuth directions φ, its applicability is restricted to samples with a special orientation of the surface.

The focus of this paper is on a straightforward and comprehensible introduction of the proposed evaluation strategies. This is achieved by the example of a simulated residual stress distribution of the surface area of a hypothetical single crystal featuring a (001) surface. To obtain a realistic scenario, the corresponding lattice parameter depth profiles were subjected to scatter with additional uncertainties for the individual data points, as also observed in experimental investigations performed on additively manufactured samples of stainless steel 316L and Inconel 718 with a pronounced mosaic structure. The presentation of these results together with the applied measurement strategy will be the subject of a separate publication. Using a similar approach, it was shown by Genzel (2001 ▸) with the example of a silicon layer with a pronounced 〈110〉 texture that lattice strain measurements along the texture poles lead to correct stress depth profiles only if the anisotropic stress factors are used for the evaluation.

The paper is structured as follows. In Section 2 the fundamental relations underlying the two stress evaluation concepts are given. In Section 3 explicit relations are derived to elucidate the influence of uncertainties of the strain-free lattice parameter *a*_0_ on the evaluated residual stress state. Simulated examples to illustrate both concepts proposed in this paper are given in Section 4. The paper is completed with some concluding remarks and a short summary.

## 2. Depth-resolved XSA in materials with single crystalline structure   

### 2.1. Fundamental relations   

**Figure 1:**
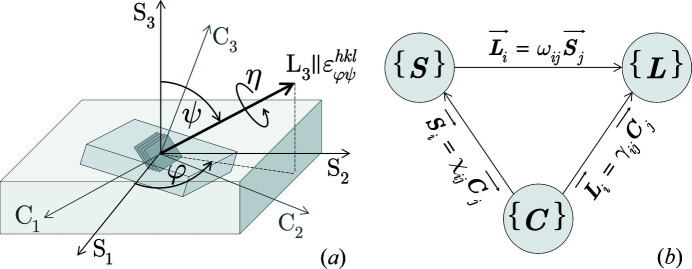
(*a*) Schematic view of the orientation relations between the different reference coordinate systems ({**S**} – sample; {**C**} – crystal; {**L**} – laboratory). (*b*) Transformation relations between the individual coordinate systems.

X-ray stress analysis is based on the measurement of lattice spacings 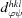 by means of Bragg’s law, 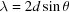, in various directions (φ, ψ) with respect to the sample reference system 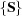 (see Fig. 1 ▸). The lattice strain 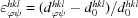 (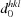 is the strain-free lattice spacing) in the measurement direction is related to the components of the stress tensor, σ_*ij*_, by the fundamental equation of XSA. Taking into account the depth dependence of the residual stress state within the sample this equation can be written in the following formal way:
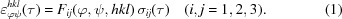
In equation (1) *F*_*ij*_ are the stress factors and τ is the information depth to which the diffraction signal can be assigned. It depends on the diffraction geometry as well as on the material’s absorption given by the linear absorption coefficient μ, which is a function of the photon energy *E*. A general formulation for τ was derived by Genzel (1994 ▸):
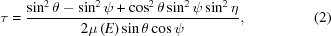
where η describes the rotation of the sample around the scattering vector 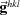. The correlation between the experimentally accessible stress depth profiles σ_*ij*_(τ) and the actual distributions in real space, σ_*ij*_(*z*), is given by
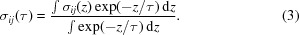
Owing to the form of equation (3) the profiles σ_*ij*_(τ) are called Laplace stress profiles. Their back transformation into the real space is difficult and currently still the subject of research. The application of the inverse numerical Laplace transform (Genzel, 1996 ▸) in many cases fails since the systems of equations to be solved are ill-conditioned (Craig & Thompson, 1994 ▸). Frequently used approaches are based on the description of the σ_*ij*_(*z*) profiles by single polynomials (Ruppersberg *et al.*, 1991 ▸), series of polynomial sections (Leverenz *et al.*, 1996 ▸) or exponentially damped functions (Hauk & Krug, 1988 ▸) which can be easily transformed into the Laplace space. The unknown parameters can be determined by least-squares fitting of the Laplace transforms to the experimental data. However, because of the empirical description of the residual stress depth distributions and the fact that different approaches provide similarly good fit results in the Laplace space, this strategy is also associated with uncertainties (Behnken & Hauk, 2001 ▸; Denks *et al.*, 2009 ▸).

The stress factors in equation (1) describe the dependence of the measurable lattice strains on the mean residual stresses. For materials featuring a pronounced crystallographic texture, which allows groups of crystallites to be treated with nearly the same orientation as a single crystal, the *F*_*ij*_ can be expressed as functions of the transformation matrices, which describe the orientation relations between the three involved reference coordinate systems (see Fig. 1 ▸), and the components of the compliance tensor ^4^ 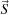. For cubic crystal symmetry one obtains for the normal components (Hauk, 1997 ▸)
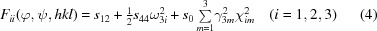
with 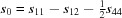. As Fig. 1 ▸(*a*) reveals, in the matrices 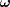 and 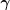 only the third-row components ω_3*i*_ and γ_3*m*_ are of interest. They represent the orientation of the measurement direction (*i.e.* the lattice strain 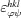) within the sample and the crystal reference system, respectively:
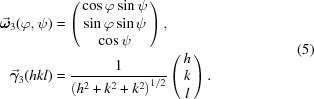
The measurement direction defined in equations (5) refers to a crystallite whose orientation relative to the sample system can be described by a set of Eulerian rotations 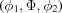 (Bunge, 1969 ▸), which are merged in the matrix
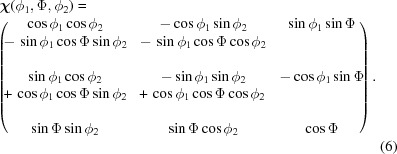
The stress factor concept can formally be applied to materials with almost random orientation distribution of the crystallites. In this case the *F*_*ij*_ become linear combinations of the ordinary diffraction elastic constants 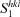 and 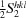: 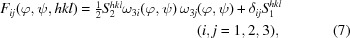
where δ_*ij*_ is the Kronecker delta and ω_3*i*_(φ, ψ) are the vector components defined by equation (5).

### 2.2. Application of the stress factor concept to materials with mosaic-like crystal structure   

#### 2.2.1. General least-squares fit approach   

**Figure 2:**
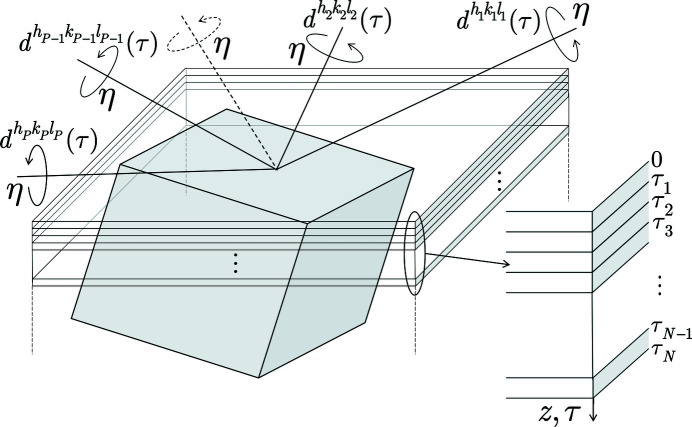
Illustration of the formalism for depth-resolved XSA in materials featuring a mosaic crystal structure using the crystallite group approach. The subscripts φ and ψ have been omitted because of the dependency 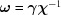.

This approach is based on an algorithm that includes the following steps (see Fig. 2 ▸):

(1) Acquisition of lattice-spacing depth profiles 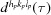 by stepwise sample rotation around the scattering vector 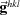 for a sufficiently large number *P* of 〈*hkl*〉 poles *p*.

(2) Analytical description of the discrete depth distributions by means of polynomial functions that are fitted to the data.

(3) Identification of overlapping depth ranges for the individual poles 〈*h*_*p*_ *k*_*p*_ *l*_*p*_〉; division of these ranges into *N* sublayers parallel to the surface.

(4) Solving equation (8) (see below) for the unknown stress components σ_*ii*_ for each sublayer *n* (*n* = 1, …, *N*); generation of σ_*ii*_(τ_*n*_) depth profiles.

(5) Back transform of the σ_*ii*_(τ) depth profiles into the real space to obtain σ_*ii*_(*z*) profiles.

Since the three matrices 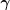, 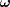 and 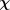 are not independent of each other, it is appropriate to replace 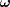 in equation (4) by 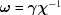. In this way it is taken into account that the angles φ and ψ for strongly pronounced textures can no longer be chosen arbitrarily but depend both on the lattice directions 〈*hkl*〉 in the crystal and on their orientation within the sample defined by 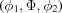. For the normal stress components σ_*ii*_ (*i* = 1, 2, 3) the system of equations to be solved for each predefined depth τ_*n*_ then becomes
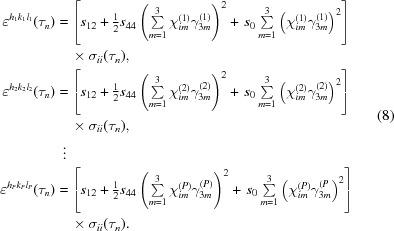
The allocation of the matrix elements of 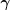 and 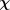 to the individual poles 〈*h*_*p*_ *k*_*p*_ *l*_*p*_〉 (*p* = 1, …, *P*) in the above system of equations is indicated by the bracketed superscripts. If equation (8) is solved for the unknown stress components σ_*ii*_ in the form given above, which reads more concisely as 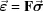 where **F** is the (*R* × *P*) matrix of the stress factors (*R* is the number of involved normal stress components σ_*ii*_, and *P* the number of measured 〈*hkl*〉 poles), a value for the strain-free lattice parameter *a*_0_ is required as input parameter to calculate the components of the strain vector 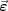 on the left-hand side.

If the residual stress state in the near-surface region can be assumed to be biaxial (*i.e.* σ_33_ ≡ 0), the proposed algorithm allows the evaluation of *a*_0_ as well. In this case the system of equations (8) is rewritten as 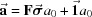 [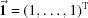 is the unity vector], which reads for each predefined depth τ_*n*_ and pole 〈*hkl*〉 (hereinafter abbreviated by *p*)
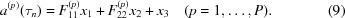
Here, 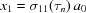, 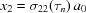 and *x*_3_ 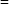 *a*_0_ are the parameters to be evaluated. The use of equation (9) and the second approach introduced in Section 2.2.2 requires the normalization of the lattice spacings *d* ^*hkl*^ to the edge length of the unit cell, *a* ^100^. Therefore, we use in the following *a* ^*hkl*^ = *d* ^*hkl*^(*h* ^2^ + *k* ^2^ + *l* ^2^)^1/2^ instead of *d* ^*hkl*^.

The uncertainties Δ_0_ of the strain-free lattice parameter *a*_0_ are known to represent a considerable source of error in XSA (Evenschor & Hauk, 1975 ▸; Dölle & Hauk, 1976 ▸). Their influence on the results obtained must therefore be carefully evaluated for each newly developed XSA method. In Section 3 the relationship between Δ_0_ and the resulting shift 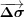 of the residual stress state is derived in explicit form for the evaluation algorithms proposed here.

#### 2.2.2. Linear regression approach   

The advantages of the formalism outlined in Section 2.2.1 are that there are no restrictions concerning the orientation of the crystallite group(s) within the sample and that the data obtained for any pole 〈*hkl*〉 can be included in the evaluation procedure. A disadvantage of this approach is the lack of clarity regarding the representation of the lattice strains versus a given parameter, as is the case for example with the 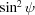 method.

**Figure 3:**
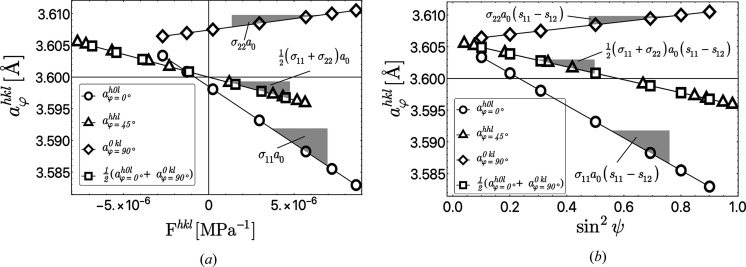
Application of the linear regression approach according to equations (11*a*)–(11*d*) to a biaxial stress state with σ_11_ = −500 MPa and σ_22_ = +100 MPa. The material used in the simulation was an austenitic steel (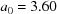
 Å); the elastic single crystal compliances *s*_*ij*_ were taken from Landoldt-Börnstein (1984 ▸). The poles 〈*hkl*〉 involved in the evaluation correspond to the poles for the azimuthal directions φ = 0, 45 and 90° in Fig. 4 ▸. See text for further details.

In the following we demonstrate that the stress factor concept, which allows for a linear regression analysis of strain data obtained from multiple reflections *hkl* by means of energy- (Klaus & Genzel, 2017 ▸; Klaus *et al.*, 2017 ▸) or angle-dispersive (Marciszko-Wiąckowska *et al.*, 2019 ▸) diffraction, can be applied also to single crystalline materials if the sample surface possesses a (001) orientation. In this case 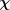 becomes the identity matrix **I**_3_, *i.e.* the coordinate systems {**S**} and {**C**} in Fig. 1 ▸ coincide. If the normalized lattice spacings 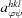 are determined under these conditions for distinct poles 〈*hkl*〉 at the azimuths 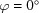 (poles 〈*h*0*l*〉), 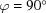 (poles 〈0*kl*〉) and 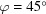 (poles 〈*hhl*〉), respectively, 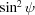 can be expressed in terms of the Miller indices:
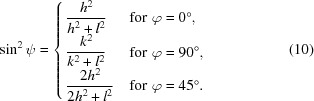
Assuming a biaxial residual stress state we obtain from equations (1) and (4) the following relations between the lattice spacings and the principal stress components for any depth τ_*n*_: 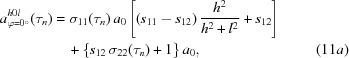


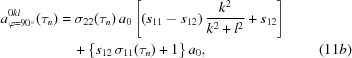


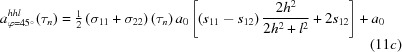


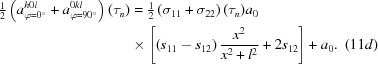
In equation (11*d*)[Disp-formula fd11d] *x* stands for *h* and *k*, which have the same value when averaging is performed over the two azimuths 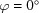 and 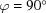. The above equations allow two different linear representations of the data (see Fig. 3 ▸). If the lattice spacings are plotted versus the stress factors (the terms in the square brackets), the slopes of regression lines fitted to the data are proportional to the product of the respective stress component and the strain-free lattice parameter *a*_0_ [Fig. 3 ▸(*a*) ▸]. In this representation the value of *a*_0_ can be determined directly from the intersection of the regression line obtained from equations (11*c*)[Disp-formula fd11c] and/or (11*d*)[Disp-formula fd11d] with the ordinate axis *F* ^*hkl*^ = 0. Note that in these plots the abscissa values of the data are different for the individual azimuthal directions 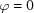, 90 and 45°, respectively, as well as for the plot of the averaged data according to equation (11*d*)[Disp-formula fd11d].

Alternatively, the data can be plotted versus 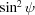 according to the relationships in equation (10) [Fig. 3 ▸(*b*) ▸]. This form of linear representation is more descriptive, since it shows the variation of the lattice spacings as a function of their orientation in the sample. The value for the strain-free lattice parameters cannot be read off directly, but it is obtained from the value of the regression line for the relations (11*c*)[Disp-formula fd11c] and (11*d*)[Disp-formula fd11d], respectively, at the position 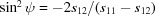. Because in both representations the plots obtained for 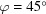 and for the data averaged over 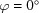 and 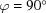 have the same slope, the data sets can be combined in a single plot and evaluated together.

## 3. Influence of the strain-free lattice parameter on residual stress evaluation for a biaxial stress state   

### 3.1. General least-squares fit approach   

**Figure 4:**
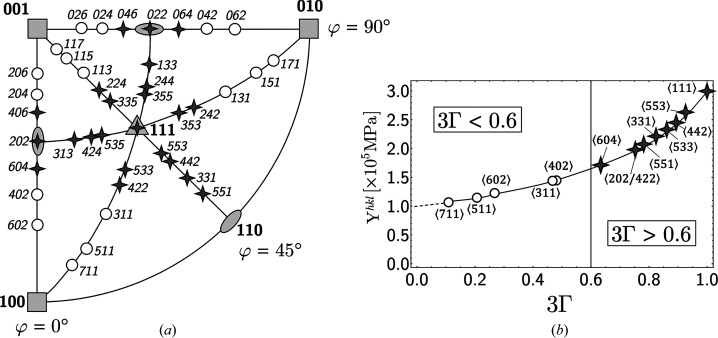
(*a*) Stereographic projection of the poles 〈*hkl*〉 used to demonstrate the two evaluation concepts introduced in this paper. (*b*) Direction-dependent Young’s modulus *Y* ^*hkl*^ for austenitic steel according to equation (16).

In the following, the depth dependence of the residual stress/strain state is omitted, since the below considerations apply to any depth τ_*n*_. Equation (9) can be rewritten as follows:
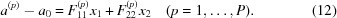
In matrix notation the above equation reads 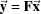, with 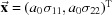, 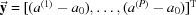 and **F** the (2 × *P*) matrix of the stress factors [see equation (8)]. We now consider two cases: (*a*) The strain-free lattice parameter *a*_0_ is known exactly and the overdetermined linear system of equations given by (12) can be solved in the usual way. (*b*) *a*_0_ is subject to an uncertainty Δ_0_ which leads to a shift 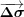 in the quantitative analysis of the residual stress state. Applying the least-squares method [see, for example, Press *et al.* (1992 ▸)] and making use of Gauss transformation yields for case (*a*)
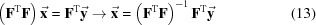
and for case (*b*)
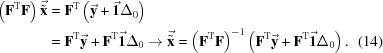
Taking the difference between equations (14) and (13), a direct connection is obtained between the relative uncertainty 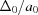 and the shift of the stress state 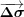: 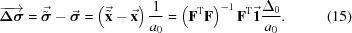
According to equation (15) 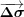 scales with 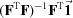. Owing to the single crystal elastic anisotropy, the stress factors 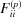, which form the matrix **F**, depend on the choice of the poles 〈*hkl*〉 included in the least-squares fitting procedure. In Fig. 4 ▸ the poles were divided into ‘hard’ and ‘soft’ directions based on the direction-dependent Young modulus *Y* ^*hkl*^, the inverse of which is proportional to the orientation factor 3Γ: 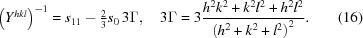
The value 3Γ = 0.6 has been chosen as the boundary between the ‘soft’ and the ‘hard’ directions. This is the crystal direction where the diffraction elastic constants for the polycrystal, 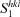 and 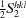, correspond to the mechanical constants, 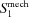 and 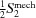, obtained by averaging over all crystal orientations. It can be seen that the hard directions are in the vicinity of the [111] pole (‘hardest’ direction) and the 〈110〉 poles, while the soft directions are found near the 〈100〉 poles (‘softest’ directions).

Table 1 ▸ reveals that the choice of the measurement directions has a strong impact on the magnitude of 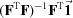. Taking into account in the analysis only the soft directions shown in Fig. 4 ▸ results in a shift of the residual stress state which is more than 30 times smaller than the shift obtained by applying the formalism exclusively to the hard directions in Fig. 4 ▸. For a relative uncertainty of 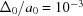 the effect on the residual stress state (*i.e.* its shift) would only be about 3 MPa in the first case, but 80 MPa in the latter case.

## 4. Linear regression approach   

In contrast to the general least-squares approach considered above, the uncertainty of *a*_0_ is of minor importance for the linear regression approach (see Fig. 3 ▸). Here the residual stress component in the azimuthal direction φ is given by the quotient of the slope *m* of the regression line and *a*_0_. Taking into account an uncertainty Δ_0_, Taylor series development yields
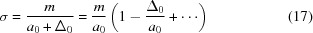
and thus
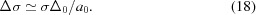
The above equation reveals that the relative uncertainty 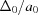 results in a shift of the stress which is about three orders of magnitude smaller than the stress value itself.

## 5. Examples   

### 5.1. Input parameters   

**Figure 5:**
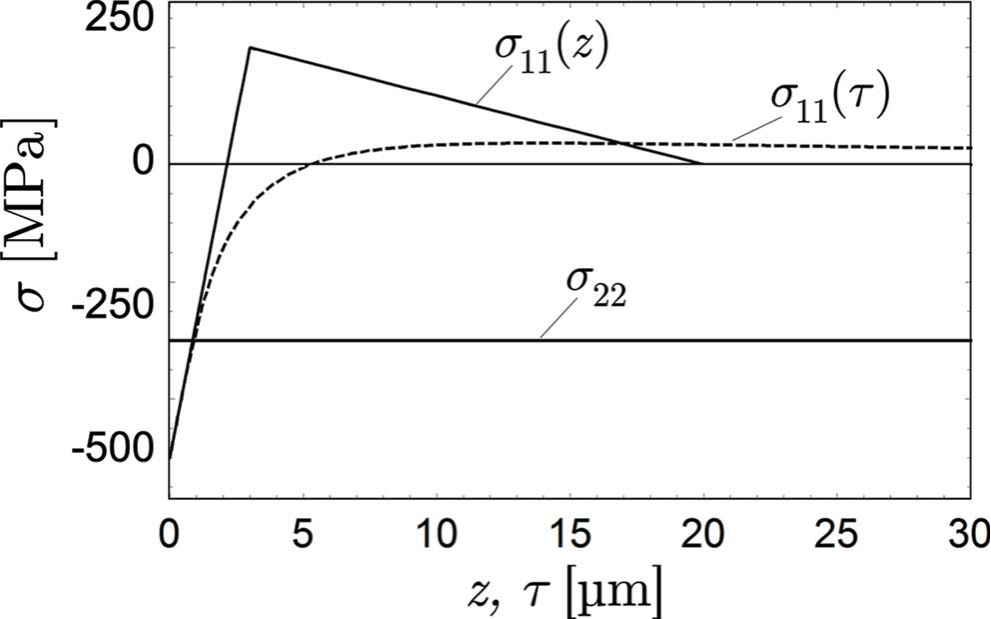
Near-surface biaxial residual stress state on which the simulations are based.

In the following the XSA strategies introduced in Section 2 are illustrated by means of a simulated example, which refers to a single crystalline austenitic steel sample featuring a (001) surface orientation. The strain-free lattice parameter 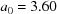
 Å is assumed to be constant in the considered depth range. The near-surface zone is subject to the biaxial residual stress state shown in Fig. 5 ▸.

**Figure 6:**
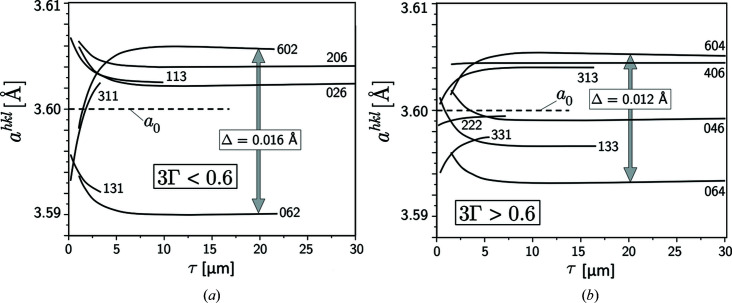
Lattice-spacing depth profiles *a* ^*hkl*^(τ) for the stress state in Fig. 5 ▸, calculated for selected 〈*hkl*〉 poles in Fig. 4 ▸ and divided into (*a*) ‘soft’ and (*b*) hard’ directions.

Normalized lattice-spacing depth profiles *a* ^*hkl*^(τ), such as would be determined by energy-dispersive diffraction, were calculated for the 〈*hkl*〉 poles shown in Fig. 4 ▸. The correlation between the positions of the diffraction line *E* ^*hkl*^ on the energy scale and the normalized lattice spacings is given by (Giessen & Gordon, 1968 ▸)
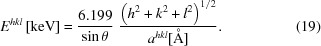
The 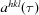 profiles in Fig. 6 ▸ were calculated for 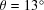. The information depth covered by the η rotation lies for the individual reflections *hkl* in the range 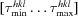. The minimum and the maximum depths correspond to incidence angles 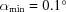 and 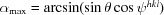, respectively, with ψ^*hkl*^ from equation (10). Depending on their orientation relative to the principal stress directions and the minimum information depth that can be achieved, the *a* ^*hkl*^(τ) profiles are characterized by more or less steep gradients near the surface and become nearly horizontal with increasing depth. Significantly larger differences occur for the soft crystal directions.

**Figure 7:**
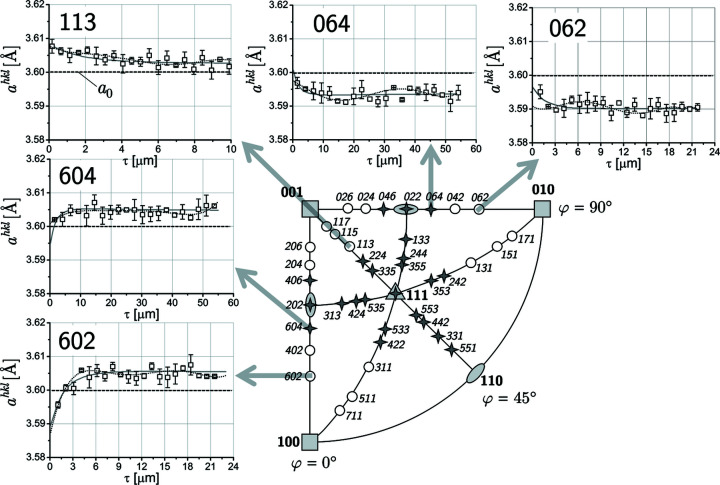
Simulated lattice-spacing depth profiles for selected 〈*hkl*〉 poles (*cf*. Fig. 6 ▸). The solid lines denote the default profiles (*cf*. Fig. 6 ▸); the dashed lines mark the curves obtained by a least-squares fit of polynomial functions of fifth order to the discrete data. Note the very different τ ranges covered for the individual 〈*hkl*〉 poles.

Data were assigned to the calculated depth profiles at discrete positions, and scatter and individual uncertainties were added, which are consistent with those measured in experimental investigations on samples from additive manufacturing. The data were fitted by polynomial functions (Fig. 7 ▸). The data base 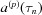 required for the application of the two evaluation concepts was generated by segmentation for depth ranges which include a sufficiently large number of lattice-spacing depth profiles (see Fig. 6 ▸).

### 5.2. Application of the general least-squares approach   

**Figure 8:**
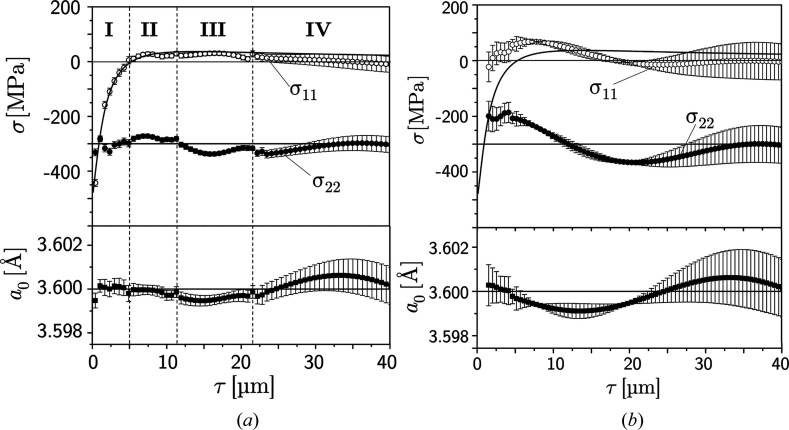
Residual stress and strain-free lattice-parameter depth profiles obtained from the general least-squares approach according to equation (9), taking into account different sets of 〈*hkl*〉 poles shown in Fig. 4 ▸. (*a*) Analysis for the poles 〈*h*0*l*〉, 〈0*kl*〉 and 〈311〉. (*b*) Analysis under exclusive consideration of the hard directions [*cf*. Fig. 4 ▸(*b*) ▸].

Figs. 6 ▸ and 7 ▸ reveal that the number of poles which can contribute to the evaluation at some depth τ_*n*_ decreases towards both very small and large depths. Consequently, very close to the surface and at large depths there will be fewer data available for the solution of the systems of equations (8) and (9), respectively, which will lead to higher uncertainties in these depth ranges. In Fig. 8 ▸(*a*), a total of 17 poles is considered, but the number of poles contributing in areas I to IV varies. The large scattering in the first micrometres below the surface is due to the fact that the high-energy diffraction lines of type {620} and {640} do not yet provide data there. At the boundaries between the individual areas, jumps occur since the 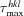 limits of several groups of reflections are reached (I/II: 202, 022, 311, 131; II/III: 402, 042, 113; III/IV: 204, 024, 602, 062). The large uncertainties in area IV are caused by the fact that there only the high-energy lines provide a contribution. Fig. 8 ▸(*b*) illustrates the situation if only the hard directions are used in the analysis. In this case, the deviations from the defaults are much larger compared with the evaluation including all (*i.e.* hard and soft) poles [see Fig. 8 ▸(*a*)]. This is similar to the findings in Section 3 for the impact of *a*_0_ uncertainties.

### 5.3. Application of the linear regression approach   

**Figure 9:**
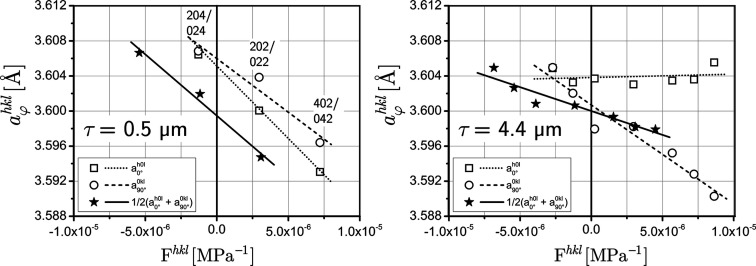
Examples for the 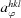
–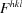 plots according to equations (11*a*), (11*b*) and (11*d*) for two information depths τ. In the left diagram, the three poles that contribute to the plots are indexed. In the right diagram, all poles 〈*h*0*l*〉 and 〈0*kl*〉 marked in Fig. 4 ▸(*a*) along the azimuthal directions φ = 0 and 90° are involved. Note the different abscissa values for the plots averaged over the two azimuths [*cf*. Fig. 3 ▸(*a*)].

While for the general least-squares approach considered in the previous section there are no restrictions with respect to the usable 〈*hkl*〉 poles, the evaluation by means of the linear regression approach is limited to the poles along the axes [100] and [010] as well as to the [110] direction. But in contrast to the general least-squares approach, the results can be presented and interpreted more clearly. Fig. 9 ▸ shows for two selected depths that only three poles at a very small depth contribute to the regression analysis, compared with seven poles in a deeper region. Consequently, the results achieved close to the surface are subject to larger uncertainties. This applies both to the slope of the regression line used to calculate the stress and to its value obtained for the averaged data set 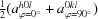 at the position *F* ^*hkl*^ = 0, which is identical to the strain-free lattice parameter *a*_0_ in the case of biaxial stress analysis.

**Figure 10:**
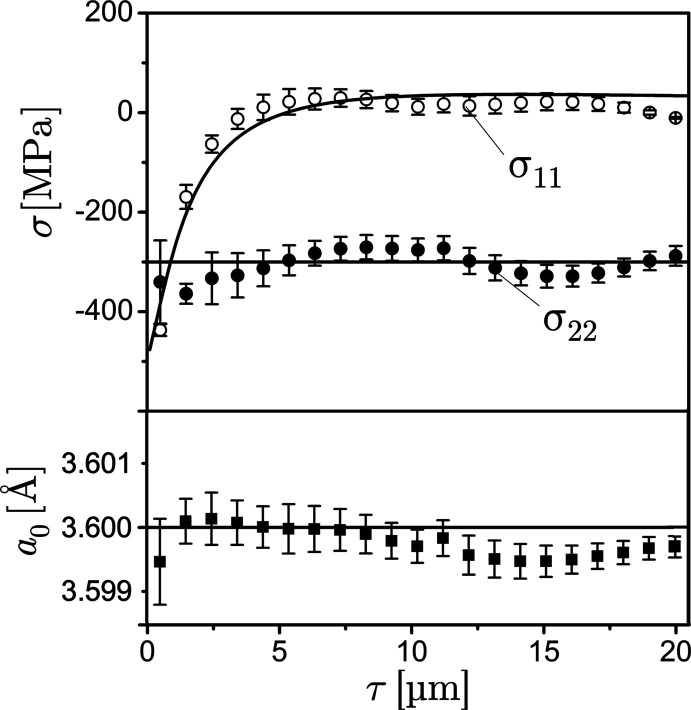
Results of the analysis obtained by the linear regression approach. Note the smaller evaluated depth range compared with Fig. 8 ▸.

The 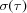 and 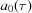 profiles determined by applying the linear regression approach to all nodes allocated in the selected τ range are shown in Fig. 10 ▸. The diagrams confirm that the regression analysis leads to results comparable to those obtained for the general least-squares approach (see Fig. 8 ▸). However, it should be noted that the total depth range considered in Fig. 10 ▸ is only half of the range shown in Fig. 8 ▸. For larger depths (not shown in the diagrams) the results become less stable, because there only three reflections (φ = 0°: 206, 406, 604; φ = 90°: 026, 046, 064) contribute to the analysis.

## 6. Concluding remarks   

Depth-resolved X-ray residual stress analysis on materials with (nearly) single crystalline structure requires a treatment which differs from that applicable to polycrystalline materials with random orientation distribution of the crystallites or weak crystallographic texture, since the number of available measurement directions is restricted to a few 〈*hkl*〉 poles and the elastic behavior of the material is anisotropic on the macroscopic length scale. In the present work, concepts are proposed on the basis of theoretical considerations which take into account the above-mentioned boundary conditions. This approach has been chosen because simulations allow one to assess the results by the deviations from the defaults and to study the influence of individual parameters (here, the direction-dependent Young modulus and the number of 〈*hkl*〉 poles involved in the evaluation).

The focus of this work is on the evaluation side. The data base used to introduce the two evaluation concepts was generated by calculating lattice-spacing depth profiles at distinct 〈*hkl*〉 poles for a biaxial residual stress state in the near-surface region of a hypothetical austenite single crystal with (001) orientation. This orientation represents a special case for the general least-squares fit approach (Section 2.2.1). In the case of any other orientation only the matrix 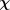 [equation (6)] changes, while the further evaluation algorithm remains unaffected.

The use of the linear regression approach in the form proposed in Section 2.2.2, on the other hand, requires a (001) orientation. We restricted the assessment of the proposed concepts to the depth profiles obtained in the Laplace space because of the challenges involved in their back transformation into the real space (see Section 2.1). The corresponding issues concern all XSA methods equally, the depth resolution of which is based on the exponential attenuation of X-rays, so that a discussion of this point would go beyond the scope of this paper.

Simulations cannot replace experiments performed on real samples featuring a characteristic microstructure. Domain size and micro-strain distributions caused by lattice defects such as dislocation (networks) and stacking faults give rise to diffraction line broadening and shifting, which superimpose the line shifts caused by macro residual stress fields. Therefore, XSA experiments make a careful separation of micro- and macrostructural influence factors mandatory. A feasible approach in this respect is to subject the diffraction lines to an additional profile analysis (Mittemeijer & Scardi, 2004 ▸).

The theoretical study presented in this paper is not based on the assumption of a specific microstructure. However, we added to the simulated data sets noise and measurement uncertainties consistent with those observed in experiments on stainless steel 316L and the nickel-base alloy Inconel 718 produced by additive manufacturing. The presentation of these results will be the subject of a separate publication, as the investigations are associated with additional issues that require detailed consideration. This implies for example the treatment of smeared-out 〈*hkl*〉 poles, which are observed when the X-ray beam illuminates near-surface areas which consist of subgrains slightly tilted or twisted relative to each other.

We see potential applications for the proposed methods in the nondestructive stress analysis of materials and components featuring a mosaic block structure consisting of crystals slightly tilted against each other in representative areas. These include materials from additive manufacturing, pronounced rolled textures or epitaxially grown layers. Extremely coarse grained materials, whose individual grains can be regarded technically as single crystals, are also conceivable. In this case, the general least-squares fit approach can be regarded as an extension of the single grain measurement method (Reimers, 1992 ▸, 1995 ▸) to enhance it by the feature of depth resolution.

## 7. Summary   

Two data evaluation concepts for depth-resolved X-ray stress analysis on materials with nearly single crystalline structure and cubic crystal symmetry have been introduced in a theoretical study. Both concepts allow the determination of residual stress gradients at the near-surface region if only a few measurement directions in the form of the 〈*hkl*〉 poles are available for the acquisition of lattice-spacing depth profiles. The proposed methods differ in their applicability and the way in which the results can be visualized. While the general least-squares approach is applicable to samples with arbitrary orientation, the linear regression approach requires samples with a (001) surface. However, the latter allows the stresses to be determined directly from the slope of plots of lattice spacings versus the stress factors or 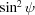. For the general least-squares approach the influence of the uncertainty of the strain-free lattice parameter *a*_0_ is shown to depend significantly on the direction-dependent Young modulus assigned to the 〈*hkl*〉 poles involved in the evaluation. Under the assumption of a biaxial residual stress state both approaches allow the refinement of *a*_0_ as a function of depth.

## Figures and Tables

**Table 1:** Influence of elastic anisotropy on the accuracy of stress determination

Poles	(**F** ^T^ **F**)^−1^ **F** ^T^ 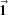 (MPa)
Soft (3Γ < 0.6)	(2.6, 2.6)^T^
Hard (3Γ > 0.6)	(80.2, 80.2)^T^
